# Genotype-phenotype correlations of Berardinelli-Seip congenital lipodystrophy and novel candidate genes prediction

**DOI:** 10.1186/s13023-020-01383-y

**Published:** 2020-04-29

**Authors:** Meng Ren, Jingru Shi, Jinmeng Jia, Yongli Guo, Xin Ni, Tieliu Shi

**Affiliations:** 1 Center for Bioinformatics and Computational Biology, and the Institute of Biomedical Sciences, School of Life Sciences, East China Normal University, Shanghai, China; 2 Beijing Key Laboratory for Pediatric Diseases of Otolaryngology, Head and Neck Surgery, MOE Key Laboratory of Major Diseases in Children, Beijing Children’s Hospital, National Center for Children’s Health, Beijing Pediatric Research Institute, Capital Medical University, Beijing, China; 3 Biobank for Clinical Data and Samples in Pediatrics, Beijing Children’s Hospital, National Center for Children’s Health, Beijing Pediatric Research Institute, Capital Medical University, Beijing, China; 4 Department of Otolaryngology, Head and Neck Surgery, Beijing Children’s Hospital, National Center for Children’s Health, Capital Medical University, Beijing, China; 5 National Center for International Research of Biological Targeting Diagnosis and Therapy, Guangxi Key Laboratory of Biological Targeting Diagnosis and Therapy Research, Collaborative Innovation Center for Targeting Tumor Diagnosis and Therapy, Guangxi Medical University, Nanning, 530021 Guangxi China

**Keywords:** Berardinelli-Seip congenital lipodystrophy, Genotype, Phenotype, Gene prediction, Protein-protein interaction, Phenotype-similarity

## Abstract

**Background:**

Berardinelli-Seip congenital lipodystrophy (BSCL) is a heterogeneous autosomal recessive disorder characterized by an almost total lack of adipose tissue in the body. Mutations in the *AGPAT2*, *BSCL2*, *CAV1* and *PTRF* genes define I-IV subtype of BSLC respectively and clinical data indicate that new causative genes remain to be discovered. Here, we retrieved 341 cases from 60 BSCL-related studies worldwide and aimed to explore genotype-phenotype correlations based on mutations of AGPAT2 and BSCL2 genes from 251 cases. We also inferred new candidate genes for BSCL through protein-protein interaction and phenotype-similarity.

**Results:**

Analysis results show that BSCL type II with earlier age of onset of diabetes mellitus, higher risk to suffer from premature death and mental retardation, is a more severe disorder than BSCL type I, but BSCL type I patients are more likely to have bone cysts. In BSCL type I, females are at higher risk of developing diabetes mellitus and acanthosis nigricans than males, while in BSCL type II, males suffer from diabetes mellitus earlier than females. In addition, some significant correlations among BSCL-related phenotypes were identified. New candidate genes prediction through protein-protein interaction and phenotype-similarity was conducted and we found that CAV3, EBP, SNAP29, HK1, CHRM3, OBSL1 and DNAJC13 genes could be the pathogenic factors for BSCL. Particularly, CAV3 and EBP could be high-priority candidate genes contributing to pathogenesis of BSCL.

**Conclusions:**

Our study largely enhances the current knowledge of phenotypic and genotypic heterogeneity of BSCL and promotes the more comprehensive understanding of pathogenic mechanisms for BSCL.

## Background

BSCL is a rare hereditary disease characterized by near-complete absence of adipose tissue in the body [1, 2]. Because whole body fat tissue lacks the ability to store fat, fat is enriched in the heart, liver and other organs [3]. This may lead to severe consequences such as diabetes mellitus, cardiomyopathy, hepatopathy, acanthosis nigricans and intellectual disability [4, 5]. In addition, affected women tend to have hirsutism, clitoromegaly, irregular menstruation, and even symptom of infertility, which may be related to hormonal changes [6, 7]. Most males with BSCL have normal reproductive capacity except a case with reported teratozoospermia [8, 9]. No conclusion has been made whether there is a gender difference in the prevalence of phenotypes shared by male and female patients.

BSCL is an autosomal recessive genetic disease with 4 different subtypes (I-IV) resulting from mutations in *AGPAT2*, *BSCL2*, *CAV1* and *PTRF* genes, respectively. Most of the cases reported so far are caused by mutations in the *BSCL2* gene (encoding protein seipin) which plays an important role in lipid homeostasis, lipid droplet assembly and adipocyte differentiation [10, 11]. Some cases have been shown to result from mutations in *AGPAT2* which encodes lysophosphatidic acid acyltransferase-β and is involved in triglyceride (triacylglycerol) biosynthesis [12, 13]. A few cases have been reported pathogenic mutations in *CAV1*, the protein of which, caveolin-1, plays a direct role in caveolar biogenesis [14, 15]; another causative gene *PTRF* encodes cavin-1 and is critical for caveolae formation and co-localizes with caveolin-1 in adipocytes [16–18]. However, some patients diagnosed as BSCL have no mutations detected in these four genes. For example, Knebel B et al. have reported that a patient with BSCL and insulin resistance has no mutations detected in these four genes but a homozygous mutation detected in the FOS promoter (C.-439, T → A) [19]. This indicates that new pathogenic mechanisms remain to be explored for BSCL.

Different BSCL subtypes have different clinical manifestations. Van Maldergem L et al. and Agarwal AK et al. studied the phenotypic heterogeneity between BSCL subtypes and discovered that BSCL type II with a higher prevalence of mental retardation and an earlier onset of diabetes mellitus appeared to be a more severe disorder than BSCL type I [3, 20]. The phenotypic heterogeneity between subtypes has gradually been confirmed with more and more clinical cases accumulated for BSCL. For example, BSCL type I develops cysts on long bones after puberty. BSCL type II is often disturbed by mental retardation. BSCL type III is associated with poor growth and short stature. BSCL type IV appears developmental delay, muscle weakness, and pyloric stenosis [21, 22]. However, each individual studies above are usually based on small sample size or regional clinical data and limit the possibility to systematically investigate the genotype-phenotype associations for BSCL.

The prevalence of BSCL is one in ten million, and the number of cases reported in the literature is about 300–500 [5]. In our study, we first collected 341 cases with BSCL from 60 studies and then inferred the genotype-phenotype associations based on 251 cases with mutations on *AGPAT2* or *BSCL2* to further illustrate the difference of phenotypic distribution in different BSCL subtypes and genders. In addition, we investigated new candidate genes based on protein-protein interaction and phenotype-similarity. Our findings deepen the understanding of the pathogenic mechanisms of BSCL and provide new insight into the discovery of additional causative genes.

## Materials and methods

### Cases collection

We searched PubMed and Google databases to collect cases. The search results were filtered solely for BSCL.

Accessibility of full text and detailed description of phenotypic features of patients with BSCL were the two important collection criteria. After manual retrieving and detailed reading of the studies, 341 cases with different ethnic background from 60 studies were collected (female: 56.9%, male: 43.1%). Since the mutations in *CAV1* or *PTRF* concern a minority of patients and the alterations in *AGPAT2* or *BSCL2* are responsible for the majority of BSCL cases (about 95% of cases) [23],our study was only focused on those cases with mutations on *AGPAT2* and *BSCL2 (*total 251) to perform genotype-phenotype analyses (Supplementary Table 1) [3, 8, 12, 20, 23–52].

### Genotype-phenotype correlations

To establish genotype-phenotype associations, we first classified patients phenotypically according to BSCL type I (*AGPAT2*) and BSCL type II (*BSCL2*) categories. Next, phenotypic information of entire 251 patients subdivided by gender in each BSCL subtype was used to explore the association between genders and phenotypes. In the end, we conducted a phenotype-phenotype correlation study to investigate whether there is a common trend between every two different phenotypes. Due to the incompleteness of phenotypical data, the number of patients varies for the different phenotypical characters [53].

All statistical analyses were performed with the software R, version 3.4.4. Student’s t-test was performed for averages. Pearson’s chi-squared test was used for two-by-two contingency tables with expected frequency > 5, and Fisher’s exact test was used for contingency tables with small expected frequency. Φ coefficient, ranging from 0 to 1, was used to measure the degree of correlation between any two phenotypes.

### New candidate gene prediction

**Fig. 1:**
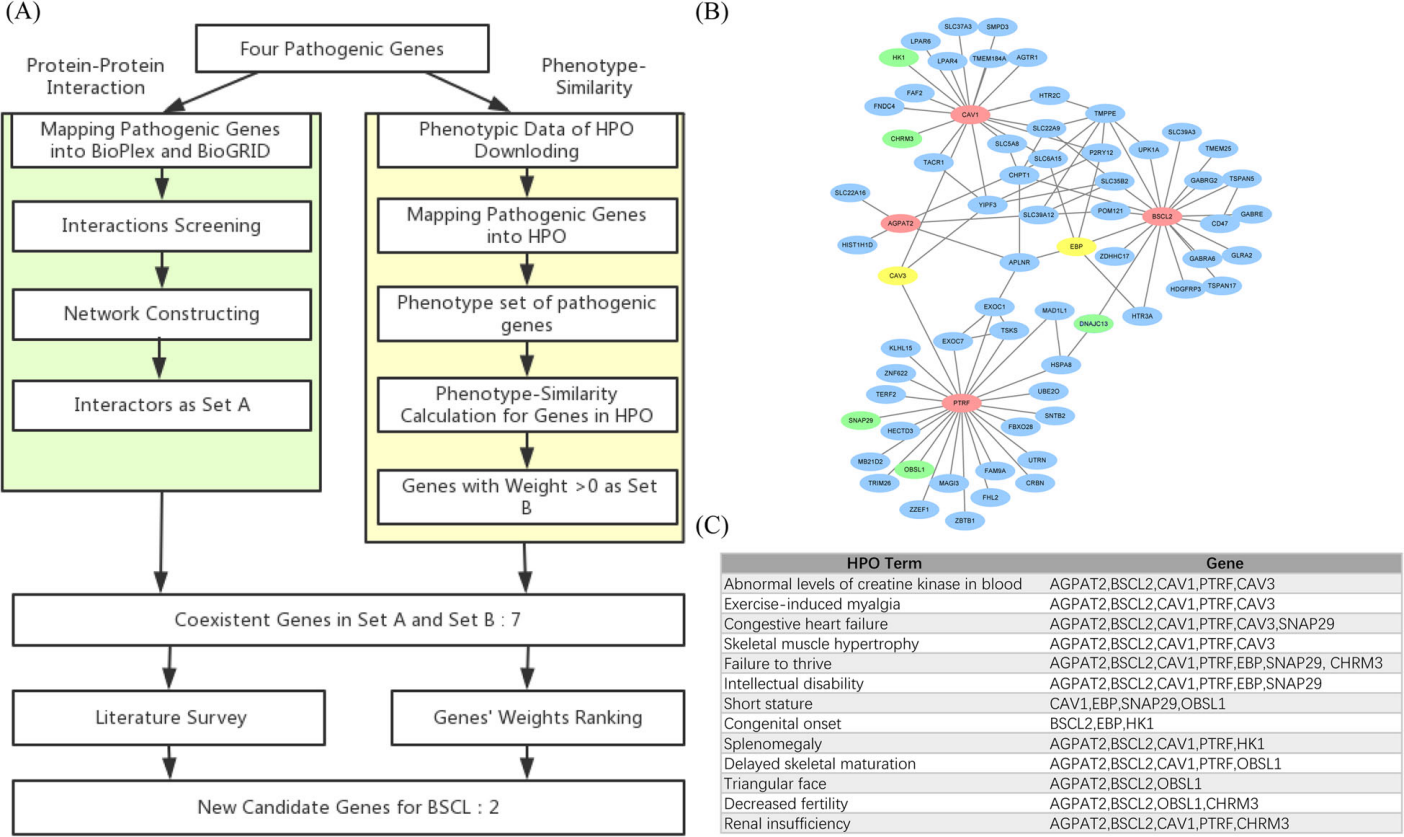
**a** A detailed flow chart for the new candidate gene prediction. The green box is the method of protein-protein interaction, and the yellow box is the method of phenotype-similarity. **b** The sub-network centered on causative proteins. Nodes in red represent the causative proteins. Nodes in green and yellow represent the BSCL-related proteins. Nodes in yellow represent the new candidate proteins we predicted. **c** BSCL-related phenotypes associated with causative genes and candidate genes

Clinical testing indicates that there are new causative genes for BSCL besides the four identified causative genes. To further explore the pathogenesis of BSCL, we conducted systematical analysis to identify new causative genes based on protein-protein interaction and phenotype-similarity. Figure 1a shows the detailed workflow for the prediction of new candidate genes.

Protein-protein interaction data from BioPlex database (version 2.0) and BioGRID database (version 3.5.165) were used in this study [54, 55]. Through mapping four proteins encoded by corresponding causative genes to BioPlex database and BioGRID database, we extracted the interaction pairs recorded by these two databases to generate a protein-protein interaction network only consisting of those causative proteins and their direct interacting partners. The set of genes that encode proteins interacting with those causative proteins was named as preliminary candidate gene set A.

Phenotypic data of genes were downloaded from Human Phenotype Ontology (HPO, http://www.human-phenotype-ontology.org, ontology version: 2018-10-09) which contains 3778 genes and 131,045 phenotypes [56]. After mapping *AGPAT2*, *BSCL2*, *CAV1* and *PTRF* genes into HPO, we obtained a unified set of phenotypes these four genes affect, named as *P*_*BSCL*_. In fact, any gene g in HPO has a corresponding phenotype set *P*_*g*_. We assumed that the more overlap between phenotypes of a gene and phenotypes related to a disease (*P*_*BSCL*_), the closer the relationship between the gene and the disease. Therefore, we compared the phenotype set *P*_*g*_ of each gene in HPO with the phenotype set *P*_*BSCL*_ and assigned each gene a weight *W*_*g*_:

$$ \mathbf{Weight}\ {\boldsymbol{W}}_{\boldsymbol{g}}=\frac{{\boldsymbol{N}}_{\boldsymbol{g}}}{{\boldsymbol{N}}_{\boldsymbol{BSCL}}} $$

*N*_*BSCL*_ represents the number of phenotypes in set *P*_*BSCL*_. *N*_*g*_ represents the number of identical phenotypes in set *P*_*BSCL*_ and *P*_*g*_. Particularly, a gene’s weight was set to 0 if its phenotype set *P*_*g*_ had no intersection with set *P*_*BSCL*_. In the end, we recorded the set of genes whose weight *W*_*g*_ > 0 as B, another preliminary candidate gene set.

We defined the genes in the intersection of these two sets A and B as BSCL-related genes. At last, we further verified the new candidate genes of BSCL through literature survey and weight ranking.

## Results

### Data presentation

A total of 341 patients with different racial background were retrieved from 60 BSCL-related studies: BSCL type I (*AGPAT2*) *n* = 83, type II (*BSCL2*) *n* = 168, type III (*CAV1*) n = 1, type IV (*PTRF*) *n* = 26, patients with unknown genotype *n* = 62, and only one patient with mutations in both *BSCL2* and *PTRF* [16]. Although this disease has been reported in different populations around the world, it appears to be more common in these three regions: Brazil (18.5%), Turkey (13.2%) and Lebanon (8.8%). The prevalence of some founder mutations and increased occurrence of endogamy probably accounts for the increased frequency of BSCL in these regions and ethnic groups [5, 21].

**Fig. 2:**
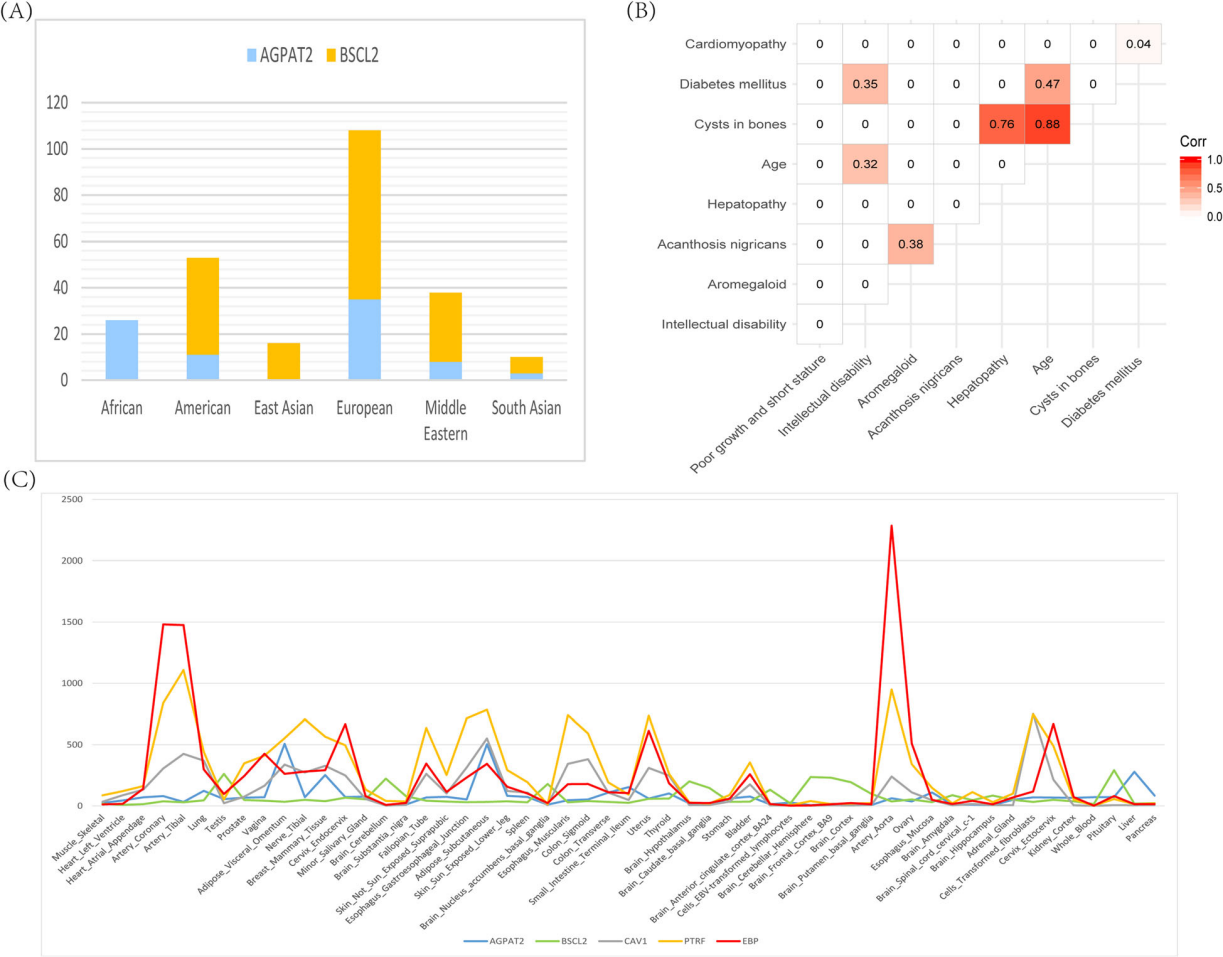
**a** A bar chart showing the differential distribution of causative genes in different ethnicities. **b** A heatmap used to show the correlation between any two phenotypes. A grid in red indicates that the relationship it represents has passed the statistical test. The value on the grid is used to measure the degree of correlation. **c** A line graph used to present the expression abundance of causative genes and gene EBP in human tissues

Due to the small number of patients with BSCL type III and type IV, only the patients with BSCL type I and type II were included for the following analyses. According to the ethnicity and geographical location, the 251 cases with BSCL type I or BSCL type II can be roughly divided into six ethnic groups: Africa, America, East Asian, European, Middle Eastern and South Asian (Fig. 2a). Surprisingly, African patients are all with mutations in *AGPAT2*, while East Asian patients are all with mutations in *BSCL2*. In each of the rest four groups, mutations in *AGPAT2* and *BSCL2* were both present, with more patients with *BSCL2* mutations than patients with *AGPAT2* mutations. This illustrates the difference in the distribution of causative genes among different ethnicities.

### Genotype-phenotype association analysis

A total of 251 patients were used to establish genotype-phenotype associations: BSCL type I (*AGPAT2*, *n* = 83) and type II (*BSCL2 n* = 168). To explore the phenotypic heterogeneity between the BSCL type I and type II, patients were firstly phenotypically classified according to BSCL types (I and II) and a difference in gender distribution between BSCL type I and BSCL type II was observed (Table 1). The number of females has much higher prevalence of BSCL type I than males, almost twice as many as males, but the ratio of female to male was balanced in BSCL type II. The age of onset of diabetes mellitus for patients in BSCL type II was significantly earlier than that in BSCL type I. It is worth noting that nearly half of the BSCL type II patients have mental development problems, while in the BSCL type I, there are very few, only 3 out of 83 cases. The major reason could be that *BSCL2* is highly expressed in many brain-related tissues. In addition, patients with premature death all belonged to BCSL type II and patients with BSCL type I are more likely to have cysts in long bones. Overall, BSCL type II with earlier onset of diabetes and higher prevalence of mental retardation and premature death appeared to be a more severe disorder than BSCL type I, which is consistent with previous studies [3, 20].

**Table 1:** Phenotypical information of entire cohort by BSCL type

	BSCL type I	BSCL type II	***P***-value
Gender(F/M)	59/24	84/84	0.0024
Mean age (y)	19.21 ± 14.72	17.45 ± 12.75	NS
Weight at birth (g)	2997.27 ± 328.78	2999.44 ± 613.61	NS
Age at onset of diabetes mellitus (y)	17.17 ± 9.95	13.24 ± 7.99	0.05
Diabetes mellitus (yes)	43%	45%	NS
Cardiomyopathy (yes)	10%	18%	NS
Hepatopathy (yes)	24%	20%	NS
Nephropathy (yes)	2%	4%	NS
Acromegaly (yes)	28%	18%	NS
Acanthosis nigricans (yes)	25%	30%	NS
Hirsutism (yes)	4%	7%	NS
Cysts in bones (yes)	16%	3%	0.01
Intellectual disability (yes)	4%	42%	< 0.0001
Poor growth and short stature (yes)	4%	7%	NS

Abbreviation: *NS* Not significant. The continuous variable is presented as mean ± standard deviation, and the categorical variable is presented as a percentage

Noticing the difference in gender distribution between BSCL type I and BSCL type 2, we further conducted a correlation study between gender and collected phenotypes (Table 2). In BSCL type I, we observed that females were at higher risk of developing diabetes mellitus and acanthosis nigricans than males. In BSCL type II, there were no other significant correlations discovered except that males were likely to suffer from diabetes mellitus earlier than females.

**Table 2:** Phenotypical information of entire cohort with BSCL type subdivided by gender

	BSCL type I			BSCL type II		
	Female	Male	***P***-value	Female	Male	***P***-value
Mean age (y)	20.13 ± 14.26	15.24 ± 16.18	NS	18.52 ± 13.76	16.42 ± 11.68	NS
Weight at birth (g)	2930 ± 303.3	3053.33 ± 366.48	NS	2822.86 ± 495.76	3081.70 ± 652.73	NS
Age at onset of diabetes mellitus (y)	16.75 ± 7.74	20.5 ± 27.58	NS	14.90 ± 8.31	10.64 ± 6.92	0.05
Diabetes mellitus (yes)	51%	25%	0.05	51%	39%	NS
Cardiomyopathy (yes)	7%	17%	NS	14%	23%	NS
Hepatopathy (yes)	7%	17%	NS	19%	23%	NS
Nephropathy (yes)	2%	4%	NS	2%	6%	NS
Acromegaly (yes)	20%	46%	NS	17%	20%	NS
Acanthosis nigricans (yes)	29%	17%	0.01	31%	31%	NS
Hirsutism (yes)	3%	4%	NS	8%	6%	NS
Cysts in bones (yes)	17%	13%	NS	2%	4%	NS
Intellectual disability (yes)	5%	0%	NS	33%	50%	NS
Poor growth and short stature (yes)	3%	4%	NS	10%	4%	NS

Abbreviation: *NS* Not significant. The continuous variable is presented as mean ± standard deviation, and the categorical variable is presented as a percentage

To investigate whether two phenotypes tend to co-exist, we performed correlation analyses among phenotypes (Fig. 2b). We discovered that the occurrence of cysts in bones and diabetes mellitus were related to age (corr = 0.88 / 0.47). We also found that there was significant correlation between cysts in bones and hepatopathy (corr = 0.76). The data showed that many people with BSCL developed diabetes mellitus during puberty and the average age of onset was 14 year old. Although some phenotypic pairs passed statistical test, their correlation coefficients were small and their relationship needs to be further examined with more data.

### New disease candidate gene prediction

Based on the protein-protein interaction, we obtained 64 preliminary candidate genes (gene set A) through mapping four proteins encoded by corresponding causative genes into BioPlex database and BioGRID database. In the HPO database, we downloaded a total of 3778 annotated genes and the corresponding 131,045 phenotypes. We obtained a unified set *P*_*BSCL*_ through mapping four causative genes into HPO. *P*_*BSCL*_ is a set with 215 phenotypes, the phenotypic set *P*_*g*_ of each gene in HPO were compared with *P*_*BSCL*_ to generate a weight for each gene. After removing the genes with a weight *W*_*g*_ = 0, a gene set B was obtained. We selected those genes in the intersection of A and B as new BSCL-related genes, *CAV3*, *EBP*, *SNAP29, HK1, CHRM3, OBSL1 and DNAJC13* met the criteria and were the candidates (Fig. 1b, c).

Among these seven potential BSCL-related genes, *CAV3* and *EBP* have higher weights, and share 12 and 9 phenotypes with causative genes, respectively. At the same time, we noted that the protein encoded by *CAV3* interacts directly with two (*CAV1,PTRF*) of the four causative proteins (Fig. 1b). It has been reported that caveolae is particularly important for normal fat transport and storage, while both *CAV1 and PTRF* are critical for the formation and stabilization of caveolae [57]. This indicates that *CAV3* could have a stronger connection with BSCL by indirectly affecting caveolae. The expression pattern of the gene *EBP* in various tissues is highly consistent with the causative genes *CAV1* and *PTRF* (Fig. 2c, The expression data of each gene was extracted from GTEx) [58]. Protein encoded by *EBP* is involved in the process of cholesterol biosynthesis and it is well known that cholesterol participates in lipid transport in human blood. Taken together, we proposed that *CAV3* and *EBP* could be high-priority candidate genes contributing to BSCL.

## Discussion

Deciphering the genotype and phenotype association can help clinicians to better conduct the diagnosis of related diseases, especially for rare diseases [52, 59]. Because of the low incidence rate, many rare diseases are not recognized by medical professional, which prohibit clinicians from accurate diagnosis of rare diseases [60–63]. In our study, we collected the largest BSCL-cohort with comprehensive genotype-phenotype information and the patients from different ethnicities included in this study covered different regions. Previous researches on the genotype-phenotype relationship of BSCL were limited to a certain ethnicity or a certain region, and the research results may only be applied to specific populations [20]. With the large cohort of different population from different geographies, we are able to systematically evaluate the associations between genotypes and phenotypes for BSCL and the results can help better understand the underlying mechanisms of the disease. In addition, we also have identified several potential new causative genes through the integrative analyses of PPIs and phenotype similarity. Among those candidates, *CAV3* and *EBP* are the most likely ones contributing to the pathogenicity of BSCL, which provide new clues for clinical diagnosis of BSCL.

Caveolin-3, encoded by *CAV3*, is a member of caveolin family distributing on the plasma membrane and caveolins are the signature proteins of caveolae [15, 64]. Studies have shown that caveolae are particularly abundant in adipocytes where caveolae seem to be essential for normal fat processing, transport, and storage. Caveolin-1, encoded by *CAV1* (a causative gene of BSCL), is also a member of this family. In this family, caveolin-3 is most closely related to caveolin-1 based on protein sequence homology with ~ 65% identity and ~ 85% similarity [65]. Capozza et al. have reported that *CAV3* null mice show more significant hyperinsulinemia and hepatic insulin resistance than wild-type mice, which demonstrates that *CAV3* plays an important role in the insulin resistance development and glucose uptake [66]. Similarly, Talukder et al. have reported that in addition to cause cardiomyopathy, *CAV3* haploinsufficiency also causes a down-regulation of glucose uptake and lipid transport in cardiomyocytes, and produces palmitate-induced insulin resistance [67]. Insulin resistance and cardiomyopathy are two typical symptoms of BSCL [68]. Collectively, above evidences further prove that *CAV3* is strongly related to the pathogenesis of BSCL.

*EBP*, encoding Delta (8)-Delta (7) sterol isomerase, is involved in the pathway cholesterol biosynthesis and mutations in *EBP* that influence the isomerase activity will significantly impair cholesterol biosynthesis [69, 70]. Cholesterol is a fundamental component of the cell membrane and is essential for maintaining membrane stability and fluidity. Especially for Caveolae membrane, cholesterol is indispensable to maintain its flask-shaped structure and ability to transport molecules [71]. Studies have shown that caveolae are particularly rich in adipocytes and are important for the normal transport and storage of fats. Based on the above evidences, we hypothesized that *EBP* could impact the caveolae of the cell membrane by affecting the biosynthesis of cholesterol, and ultimately affect the adipocytes to cause lipodystrophy.

For patients with severe symptoms, BSCL can lead to premature death. Lima JG et al. conducted a research on this phenomenon and discovered that the lifespan of patients, suffering from premature death, could be cut by 30 or more years [24]. Of the 251 patients we analyzed, premature death was only found in BSCL type II and 21 patients (12 female and 9 male) died. The causes of deaths were divided into three major groups: renal failure (6 patients, 29%), heart failure (5 patients, 24%), and other causes (sepsis, three patients; acute pancreatitis, three patients; liver cirrhosis, two patients; gastrointestinal bleeding, two patients).

Although our bioinformatic approach has expended our understanding of BSCL disease, this study does have certain limitations. For example, the recording phenotypes of cases from different articles are inconsistent. In addition, patient’s symptoms always occur with ageing, so phenotypic information in adults is more abundant than in children. In order to explore whether the observations here would be seriously affected by the proportion of adults and children, we performed analysis only in the adult age group and found that the results were basically consistent with the results of analysis with all samples, except that the age of onset of diabetes was not significantly different between the two subtypes (Supplementary Table 2). All in all, there is still an urgent need for large-scale, well-designed research to further improve our understanding of BSCL.

## Conclusions

In summary, patients with *BSCL2* mutations show earlier age of onset of diabetes mellitus, higher risk to suffer from premature death and mental retardation and are more severe than patients with *AGPAT2* mutations. Females are at higher risk of developing diabetes mellitus and acanthosis nigricans than males in BSCL type I, while males are likely to suffer from diabetes mellitus earlier than females in BSCL type II. Furthermore, new candidate genes (*CAV3* and *EBP*) have been predicted through protein interactions and phenotype similarity. Our findings advance the knowledge of the mechanisms behind BSCL and provide an alternative way for the study of rare diseases.

## Supplementary information

**Additional file 1: Table S1.** The cases with AGPAT2 or BSCL2 mutations we collected were from these articles in the table. **Table S2.** Phenotypical information of adult patients by BSCL type.

## Data Availability

The data that support the findings of this study is from secondary data sources and has been cited in the manuscript or has been provided in the supplementary Table 1.

## Abbreviations

BSCL: Berardinelli-Seip congenital lipodystrophy
HPO: Human Phenotype Ontology
