# Supplementary material for: Genotype-phenotype correlations of Berardinelli-Seip congenital lipodystrophy and novel candidate genes prediction
Source: Orphanet J Rare Dis. 2020 Apr 29;15:108. doi: 10.1186/s13023-020-01383-y (PMC7191718; doi:10.1186/s13023-020-01383-y)
Supplement: Supplementary file 1 — Additional file 1: Table S1. The cases with AGPAT2 or BSCL2 mutations we collected were from these articles in the table. Table S2. Phenotypical information of adult patients by BSCL type. [file 13023_2020_1383_MOESM1_ESM.pdf]

Table 1 The cases with AGPAT2 or BSCL2 mutations we collected were from these articles in the table.

Author (year)	Number of cases
Van Maldergem L.2002(1)	58
Lima JG.2018(2)	12
Agarwal AK.2003(3)	17
Agarwal AK.2002(4)	20
Haghighi A.2015(5)	10
Akinci B.2016(6)	27
Araujo-Vilar D.2015(7)	6
Fu M.2004(8)	29
Opri R.2016(9)	3
Barra CB.2011(10)	4
Ben Turkia H.2009(11)	1
Bhayana S.2002(12)	1
Debray FG.2013(13)	1
Huang HH.2010(14)	1
Shirwalkar HU.2008(15)	1
Ebihara K.2004(16)	3
Nishiyama A.2009(17)	2
Jin J.2007(18)	1
Friguls B.2009(19)	1
Ferraria N.2013(20)	3
Haghighi A.2012(21)	2
Hann G.2013(22)	1
Jeninga EH.2012(23)	1
Metwalley KA.2014(24)	1
Solanki M.2008(25)	2
Victoria B.2010(26)	1
Simsir IY.2017(27)	1
Purizaca-Rosillo N.2017(28)	6
Su X.2017(29)	3
Chen R.2017(30)	2
Ponte CMM.2018(31)	7
Lima JG.2017(32)	11
Dantas de Medeiros JL.2018(33)	11
Gonzalo MM.2017(34)	1

1. Van Maldergem L, Magre J, Khallouf TE, Gedde-Dahl T, Jr., Delepine M, Trygstad O, et al. Genotype-phenotype relationships in Berardinelli-Seip congenital lipodystrophy. J Med Genet. 2002;39(10):722-33.
2. Lima JG, Nobrega LHC, Lima NN, Dos Santos MCF, Silva PHD, Baracho MFP, et al. Causes of death in patients with Berardinelli-Seip congenital generalized lipodystrophy. PLoS One. 2018;13(6):e0199052.
3. Agarwal AK, Simha V, Oral EA, Moran SA, Gorden P, O'Rahilly S, et al. Phenotypic and genetic heterogeneity in congenital generalized lipodystrophy. J Clin Endocrinol Metab. 2003;88(10):4840-7.
4. Agarwal AK, Arioglu E, De Almeida S, Akkoc N, Taylor SI, Bowcock AM, et al. AGPAT2 is mutated in congenital generalized lipodystrophy linked to chromosome 9q34. Nat Genet. 2002;31(1):21-3.
5. Haghighi A, Kavehmanesh Z, Haghighi A, Salehzadeh F, Santos-Simarro F, Van Maldergem L, et al. Congenital generalized lipodystrophy: identification of novel variants and expansion of clinical spectrum. Clin Genet. 2016;89(4):434-41.
6. Akinci B, Onay H, Demir T, Ozen S, Kayserili H, Akinci G, et al. Natural History of Congenital Generalized Lipodystrophy: A Nationwide Study From Turkey. J Clin Endocrinol Metab. 2016;101(7):2759-67.
7. Araujo-Vilar D, Sanchez-Iglesias S, Guillin-Amarelle C, Castro A, Lage M, Pazos M, et al. Recombinant human leptin treatment in genetic lipodystrophic syndromes: the long-term Spanish experience. Endocrine. 2015;49(1):139-47.
8. Fu M, Kazlauskaite R, Baracho Mde F, Santos MG, Brandao-Neto J, Villares S, et al. Mutations in Gng3lg and AGPAT2 in Berardinelli-Seip congenital lipodystrophy and Brunzell syndrome: phenotype variability suggests important modifier effects. J Clin Endocrinol Metab. 2004;89(6):2916-22.
9. Opri R, Fabrizi GM, Cantalupo G, Ferrarini M, Simonati A, Dalla Bernardina B, et al. Progressive Myoclonus Epilepsy in Congenital Generalized Lipodystrophy type 2: Report of 3 cases and literature review. Seizure. 2016;42:1-6.
10. Barra CB, Savoldelli RD, Manna TD, Kim CA, Magre J, Porta G, et al. [Genetic and metabolic description of five patients with Berardinelli-Seip syndrome]. Arq Bras Endocrinol Metabol. 2011;55(1):54-9.
11. Ben Turkia H, Tebib N, Azzouz H, Abdelmoula MS, Ben Chehida A, Hubert P, et al. [Congenital generalized lipodystrophy: a case report with neurological involvement]. Arch Pediatr. 2009;16(1):27-31.
12. Bhayana S, Siu VM, Joubert GI, Clarson CL, Cao H, Hegele RA. Cardiomyopathy in congenital complete lipodystrophy. Clin Genet. 2002;61(4):283-7.
13. Debray FG, Baguette C, Colinet S, Van Maldergem L, Verellen-Dumouin C. Early infantile cardiomyopathy and liver disease: a multisystemic disorder caused by congenital lipodystrophy. Mol Genet Metab. 2013;109(2):227-9.
14. Huang HH, Chen TH, Hsiao HP, Huang CT, Wang CC, Shiau YH, et al. A Taiwanese boy with congenital generalized lipodystrophy caused by homozygous Ile262fs mutation in the BSCL2 gene. Kaohsiung J Med Sci. 2010;26(11):615-20.
15. Shirwalkar HU, Patel ZM, Magre J, Hilbert P, Van Maldergem L, Mukhopadhyay

RR, et al. Congenital generalized lipodystrophy in an Indian patient with a novel mutation in BSCL2 gene. J Inherit Metab Dis. 2008;31 Suppl 2:S317-22.
16. Ebihara K, Kusakabe T, Masuzaki H, Kobayashi N, Tanaka T, Chusho H, et al. Gene and phenotype analysis of congenital generalized lipodystrophy in Japanese: a novel homozygous nonsense mutation in seipin gene. J Clin Endocrinol Metab. 2004;89(5):2360-4.
17. Nishiyama A, Yagi M, Awano H, Okizuka Y, Maeda T, Yoshida S, et al. Two Japanese infants with congenital generalized lipodystrophy due to BSCL2 mutations. Pediatr Int. 2009;51(6):775-9.
18. Jin J, Cao L, Zhao Z, Shen S, Kiess W, Zhi D, et al. Novel BSCL2 gene mutation E189X in Chinese congenital generalized lipodystrophy child with early onset diabetes mellitus. Eur J Endocrinol. 2007;157(6):783-7.
19. Friguls B, Coroleu W, del Alcazar R, Hilbert P, Van Maldergem L, Pintos-Morell G. Severe cardiac phenotype of Berardinelli-Seip congenital lipodystrophy in an infant with homozygous E189X BSCL2 mutation. Eur J Med Genet. 2009;52(1):14-6.
20. Ferraria N, Pedrosa C, Amaral D, Lopes L. Berardinelli-Seip syndrome: highlight of treatment challenge. BMJ Case Rep. 2013;2013.
21. Haghighi A, Razzaghy-Azar M, Talea A, Sadeghian M, Ellard S, Haghighi A. Identification of a novel nonsense mutation and a missense substitution in the AGPAT2 gene causing congenital generalized lipodystrophy type 1. Eur J Med Genet. 2012;55(11):620-4.
22. Hann G, Salam A, Kapila P. An incidental finding of striking muscular hypertrophy leading to a diagnosis of Berardinelli Seip Congenital Lipodystrophy. Arch Dis Child. 2013;98(12):933.
23. Jeninga EH, de Vroede M, Hamers N, Breur JM, Verhoeven-Duif NM, Berger R, et al. A Patient with Congenital Generalized Lipodystrophy Due To a Novel Mutation in BSCL2: Indications for Secondary Mitochondrial Dysfunction. JIMD Rep. 2012;4:47-54.
24. Metwalley KA, Farghaly HS. Berardinelli-Seip syndrome type 1 in an Egyptian child. Indian J Hum Genet. 2014;20(1):75-8.
25. Solanki M, Patil SS, Baweja DK, Noorani H, Pk S. Talon cusps, macrodontia, and aberrant tooth morphology in Berardinelli-Seip syndrome. Oral Surg Oral Med Oral Pathol Oral Radiol Endod. 2008;105(1):e41-7.
26. Victoria B, Cabezas-Agricola JM, Gonzalez-Mendez B, Lattanzi G, Del Coco R, Loidi L, et al. Reduced adipogenic gene expression in fibroblasts from a patient with type 2 congenital generalized lipodystrophy. Diabet Med. 2010;27(10):1178-87.
27. Simsir IY, Yurekli BS, Saygili F, Altay C, Akinci B. First metreleptin treatment for generalized lipodystrophy in Turkey. Diabetes Obes Metab. 2017;19(2):299-301.
28. Purizaca-Rosillo N, Mori T, Benites-Condor Y, Hisama FM, Martin GM, Oshima J. High incidence of BSCL2 intragenic recombinational mutation in Peruvian type 2 Berardinelli-Seip syndrome. Am J Med Genet A. 2017;173(2):471-8.
29. Su X, Lin R, Huang Y, Sheng H, Li X, Ting TH, et al. Clinical and Mutational Features of Three Chinese Children with Congenital Generalized Lipodystrophy. J Clin Res Pediatr Endocrinol. 2017;9(1):52-7.

30. Chen R, Yuan X, Wang J, Zhang Y. Clinical and molecular characterization of two Chinese patients with Type 2 congenital generalized lipodystrophy. Gene. 2017;637:57-62.
31. Ponte CMM, Fernandes VO, Gurgel MHC, Vasconcelos I, Karbage L, Liberato CBR, et al. Early commitment of cardiovascular autonomic modulation in Brazilian patients with congenital generalized lipodystrophy. BMC Cardiovasc Disord. 2018;18(1):6.
32. Lima JG, Nobrega LHC, Lima NN, Dos Santos MCF, Baracho MFP, Winzenrieth R, et al. Normal bone density and trabecular bone score, but high serum sclerostin in congenital generalized lipodystrophy. Bone. 2017;101:21-5.
33. Dantas de Medeiros JL, Carneiro Bezerra B, Brito de Araujo TA, Craveiro Sarmento AS, de Azevedo Medeiros LB, Peroni Gualdi L, et al. Impairment of respiratory muscle strength in Berardinelli-Seip congenital lipodystrophy subjects. Respir Res. 2018;19(1):173.
34. Gonzalo MM, Estefania CV. Congenital Generalized Lipodystrophy Type 2 in a Patient From a High-Prevalence Area. J Endocr Soc. 2017;1(8):1012-4.

Table 2 Phenotypical information of adult patients by BSCL type.

	BSCL type I	BSCL type II	P-value
Gender(F/M)	31/7	33/29	0.0080
Mean age (y)	31.24±12.61	30.65±8.52	NS
Weight at birth (g)	2852.50±247.03	3141.33±597.05	NS
Age at onset of diabetes mellitus (y)	19.71±9.61	16.52±8.21	NS
Diabetes mellitus (yes)	32%	24%	NS
Cardiomyopathy (yes)	5%	6%	NS
Hepatopathy (yes)	5%	7%	NS
Nephropathy (yes)	3%	1%	NS
Acromegaly (yes)	13%	7%	NS
Acanthosis nigricans (yes)	24%	8%	NS
Hirsutism (yes)	3%	0%	NS
Cysts in bones (yes)	32%	3%	0.0023
Intellectual disability (yes)	5%	46%	0.0001
Poor growth and short stature (yes)	5%	2%	NS

Abbreviation: NS, not significant. The continuous variable is presented as mean ± standard deviation, and the categorical variable is presented as a percentage.
